# Health Status of US Patients With One or More Health Conditions: Using a Novel Electronic Patient-reported Outcome Measure Producing Single Metric Measures

**DOI:** 10.1097/MLR.0000000000001919

**Published:** 2023-09-13

**Authors:** Xin Zhang, Karin M. Vermeulen, Paul F.M. Krabbe

**Affiliations:** Department of Epidemiology, University Medical Center Groningen, University of Groningen, Groningen, The Netherlands

**Keywords:** comorbidity, health status, multimorbidity, patient-reported outcome measure

## Abstract

**Background:**

Most existing research studying health status impacted by morbidity has focused on a specific health condition, and most instruments used for measuring health status are neither patient-centered nor preference-based. This study aims to report on the health status of patients impacted by one or more health conditions, measured by a patient-centered and preference-based electronic patient-reported outcome measure.

**Methods:**

A cross-sectional study was conducted among patients with one or more health conditions in the United States. A novel generic, patient-centered, and preference-based electronic patient-reported outcome measure: Château Santé-Base, was used to measure health status. Individual health state was expressed as a single metric number (value). We compared these health-state values between sociodemographic subgroups, between separate conditions, between groups with or without comorbidity, and between different combinations of multimorbidity.

**Results:**

The total sample comprised 3913 patients. Multimorbidity was present in 62% of the patients. The most prevalent health conditions were pain (50%), fatigue/sleep problems (40%), mental health problems (28%), respiratory diseases (22%), and diabetes (18%). The highest (best) and lowest health-state values were observed in patients with diabetes and mental health problems. Among combinations of multimorbidity, the lowest values were observed when mental health problems were involved, the second lowest values were observed when fatigue/sleep problems and respiratory diseases coexisted.

**Conclusions:**

This study compared health status across various single, and multiple (multimorbidity and comorbidity) health conditions directly, based on single metric health-state values. The insights are valuable in clinical practice and policy-making.

Traditionally, health outcome measurement is based primarily on objective clinical indicators such as blood pressure, mortality, or survival.^1^ However, these clinical indicators do not capture a full picture of a population’s health.^2^ In addition to clinical measures, subjective indicators such as quality of life, health-related quality of life, health status, well-being, and patients’ experiences have become important in health outcome measurement.^3^ In this study, we focus on measuring patients’ perceived health status and use the term “health status.”

Morbidity is associated with reduced health status in multiple health domains including physical functions, mental health, and social functioning.^4–6^ Effective health outcome measures are needed to reflect patients’ perceived health status. Patient-reported outcome measures are a special category of health outcome measures. Patient-reported outcome measures include any assessment coming directly from patients, without interpretation by physicians or others, about how they function or feel in relation to their health status.^7^ In a previous paper, we introduced a novel generic electronic patient-reported outcome measure (ePROM) called Château Santé-Base (CS-Base).^8^

CS-Base is a patient-centered, preference-based, generic ePROM. It was fully patient-centered in its development and construction. Ideally, a generic outcome measure should capture health domains relevant to most patients across the spectrum of health care, and include basic domains that address the concept of health. To meet these requirements, in a previous study, 2256 patients with a wide range of health conditions were asked to select the most important items from a list of 47 candidate items, which were selected from existing generic preference-based measures. The 12 most important items according to the patients, were included in the CS-Base.^8^ Another innovative characteristic of CS-Base is its special measurement framework, entailing a novel preference-based measurement model.^9^ Unlike questionnaire-based instruments [eg, 36-Item Short Form Survey (SF-36),^10^ Quality of Life Questionnaire Core 30 (QLQ-C30)],^11^ which measure the intensity of separate health domains through a bundle of items, the preference-based CS-Base not only measure the intensity of separate health domains but also assigns weights to separate health domains. These weights ultimately generate a single metric number (“value”) reflecting the overall quality of a health state.^12^

The impact of morbidity on health status has been investigated in the past but mainly by health outcome measures which are neither patient-centered nor preference-based. Moreover, previous studies have mainly focused on a specific disease or health complaint but seldom covered a wide range of disease or health complaints. This paper aims to report health status of patients with one or more diseases or health complaints measured by the CS-Base ePROM.

## METHODS

### Sample

We conducted a population-based cross-sectional study. Respondents were patients (18 y and older) in the US registered in the panel of a market research company (Dynata) based in Rotterdam, The Netherlands. Dynata distributed an online survey for our study to patients via their system. Data were collected in December 2020 and February 2022. The patients’ sociodemographic data, disease, or health complaints were provided by Dynata. The sample was nationally representative for age, sex, and region.

### Health Outcomes

Patients were requested to self-report their diseases or health complaints (they could report multiple if they have) using Dynata’s classification list, including 14 diseases or health complaints: respiratory diseases, diabetes, eczema, gastrointestinal diseases, heart disease, cancer, rheumatism, stroke, epilepsy, pain, fatigue/sleep problems, mental health problems, hearing or vision loss, and other diseases. The term “health condition” is used to refer to various diseases and health complaints in this study. It is a broad concept that includes all diseases, lesions, disorders, or nonpathologic conditions that normally receive medical treatment.^13^ The term “multimorbidity” is used to indicate the presence of 2 or more health conditions without a specification of an index or primary health condition.^14,15^ “Comorbidity” refers to the existence of additional health issues alongside a primary or index condition.^16^

The health status of patients was measured by the preference-based CS-Base ePROM. It comprises 12 health items: mobility, vision, hearing, cognition, mood, anxiety, pain, fatigue, social functioning, daily activities, self-esteem, and independence. Each item is specified at 4 levels (1, 2, 3, 4). Level 1 is regarded as optimal, and the other 3 levels have some degree of problems [Supplementary Digital Content (SDC) 1, http://links.lww.com/MLR/C705]. CS-Base operated via an innovative mobile app (www.chateau-sante.info): HealthSnApp, (patent pending). Using CS-Base in the app, patients first performed a descriptive task (Task 1) describing their own health status based on the 12 items. This task generates an overall health state expressed as 12 digits (eg, 111213411121), with each digit indicating the level assessed on the 12 CS-Base items. Patients then proceeded to Task 2, which employs a preference-based task called Drop-Down (SDC 1, Supplemental Digital Content 1, http://links.lww.com/MLR/C705) (X.Z. and P.F.M.K., unpublished data, Sep. 1, 2023). Based on the data collected by the preference-based task, weights of each level of the 12 items can be estimated. Subsequently, a single metric value was computed for a health state, called health-state value. The values ranged from 0.0 to 1.0, 1.0 indicates the full health state (111111111111, it should be distinguished from the best health, which refers to the best state in a dataset but may not have all items at level 1), 0.0 indicates the worst health state (444444444444). The computation of values are explained in SDC 2 (Supplemental Digital Content 2, http://links.lww.com/MLR/C706).

### Analysis

Frequencies and percentages were used to describe the distribution of patients in subgroups of sociodemographic characteristics, health conditions, and levels of items reported on CS-Base. Means were used to describe age and health-state values. We computed mean health-state values for each separate condition on 4 groups: G1—*single condition*, G2—*single (specific) condition with comorbidity*, G3—*absence of a specific condition but with miscellaneous other conditions*, G4—*presence of a specific condition either with or without comorbidity*. The single condition in G1 is considered as the index condition in G2 and G4, G3 is absence of the single specific condition indicated in G1.

We compared mean health-state values between sociodemographic subgroups, between separate conditions, between groups with or without comorbidity, and between combinations of multimorbidity. A *t* test was used to explore the differences in mean health-state values between males and females, between with or without comorbidity. Analysis of variance was used to test differences in mean values between subgroups of age, education, and ethnicity. The Spearman correlation was computed to assess the relationship between the numbers of conditions and mean values. The Fisher exact test was used to test the difference between age groups (divided between 18–57 and >57 y) regarding education levels and the number of health conditions. To analyze and visualize the results, we used the following software packages: Stata 17.0, R studio 1.4, and CorelDraw 22.0.

## RESULTS

### Sociodemographic Characteristics

The final sample for analysis comprised 3913 patients. The mean age of the total sample was 46 years, ranging from 18 to 94 years (Table 1). There were 2163 (55%) female patients, 1601 (50%) patients had a high education level (more than secondary school). A larger proportion of patients had higher education (more than secondary school) within older age groups (51% within the group above 57 y) than within the younger groups (37% within the group 18–57 y) (*P*<0.001, Table 2). Most of the patients were White Americans (77%).

**TABLE 1:** The Number of Respondents and Mean Château Santé-Base Values Per Subgroup for Sociodemographic Factors and Different Numbers of Health Conditions (N=3913)

Subgroups	n (%)	Mean value (SD)
Total sample	3913 (100)	0.83 (0.15)
Sex	3906 (100)	
Female	2163 (55)	0.81 (0.16)
Male	1743 (45)	0.86 (0.15)
Age group (y)	3907 (100)	
18–27	636 (16)	0.80 (0.16)
28–37	819 (21)	0.81 (0.17)
38–47	684 (17)	0.82 (0.17)
48–57	596 (15)	0.81 (0.16)
58–67	623 (16)	0.87 (0.13)
68–77	453 (12)	0.90 (0.11)
≥78	96 (2)	0.92 (0.08)
Ethnicity	3864 (99)	
Asian/Asian American	129 (3)	0.86 (0.14)
Black/African American	366 (9)	0.85 (0.16)
Hispanic or Latino American	225 (6)	0.79 (0.18)
Native American/Inuit/Alaskan	61 (2)	0.87 (0.14)
Native Hawaiian/Pacific Islander	24 (1)	0.79 (0.19)
White American/Caucasian	3021 (77)	0.83 (0.15)
Other	38 (1)	0.78 (0.17)
Education	3218 (82)	
More than secondary school	1601 (41)	0.87 (0.14)
Secondary school graduate	1356 (35)	0.81 (0.16)
Less than secondary school	261 (7)	0.79 (0.17)
No. conditions	3913 (100)	
1	1488 (38)	0.90 (0.12)
2	1054 (27)	0.84 (0.13)
3	698 (18)	0.79 (0.15)
4	372 (10)	0.74 (0.16)
5	166 (4)	0.71 (0.18)
≥6	135 (3)	0.67 (0.22)

*P*<0.001 for all subgroups.

**TABLE 2:** Education Level and Number of Health Conditions by Age Groups (N=3907)

	Age group [n (%)]	
Factors	18–57 y	>57 y
Education level		
More than secondary school^*^	1000 (37)	598 (51)
Secondary school graduate^*^	890 (33)	463 (40)
Less than secondary school^*^	211 (8)	50 (4)
Missing^*^	634 (23)	61 (5)
No. conditions		
1^*^	967 (35)	519 (44)
2	728 (27)	323 (28)
3^*^	522 (19)	175 (15)
4^*^	277 (10)	95 (8)
5^*^	131 (5)	35 (3)
≥6^*^	110 (4)	25 (2)

The 57-year age break was based on the values of all age groups; the values for the 3 older age groups (58–67, 68–77, ≥78 y) are higher than younger age groups.

* Significant difference between age groups (*P*<0.05).

### Health Conditions

**FIGURE 1:**
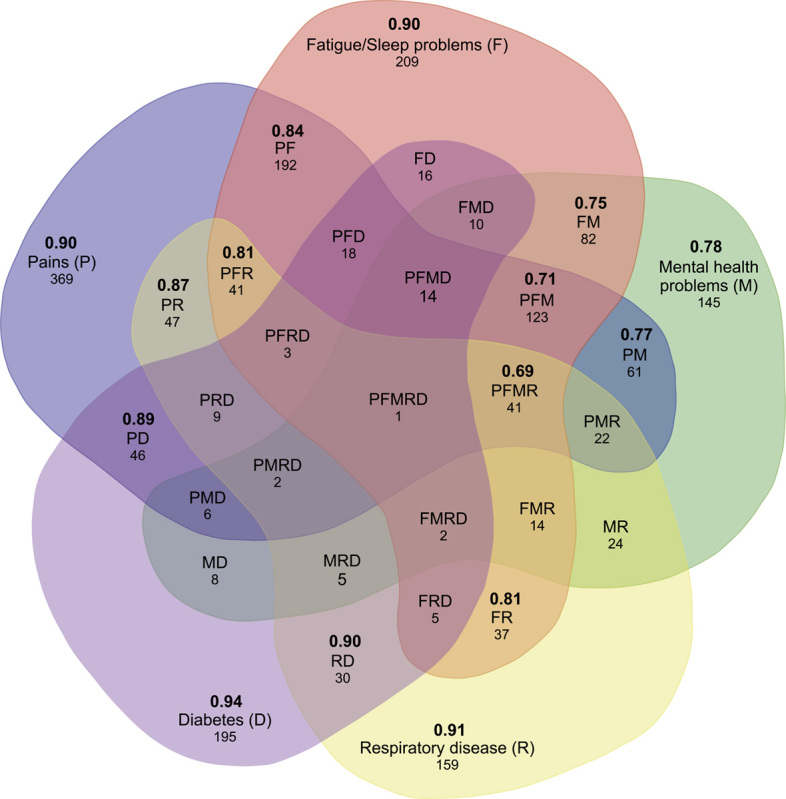
Venn diagram presenting the relationships between the top 5 health conditions. Each condition is represented by each of the 5 main sections in 5 colors. The outer layer denotes the number of respondents with this single condition. The inner layers are intersections indicating respondents suffering from 2 or more of these 5 conditions. The figures under the label of conditions represent the number of respondents for each single condition or specific combination of multimorbidity. The figures (bold) above the label of conditions represent the mean health-state values of each single condition and specific combination of multimorbidity (N≥30).

All the 3913 patients reported one or more of 14 conditions. The majority (1957, 50%) reported pain, followed by fatigue/sleep problems (1578, 40%), mental health problems (1104, 28%), respiratory diseases (855, 22%), and diabetes (704, 18%). These 5 most frequently reported conditions are referred to as the “top 5” in this paper. The majority of patients (62%) reported suffering from multimorbidity (Table 1). For the top 5, the number of patients in a single condition and combinations of multimorbidity are shown in a Venn diagram (Fig. 1). Older patients seemed to report less multimorbidity than younger patients. A larger proportion of older patients reported a single condition (44% within group >57 y) in comparison to younger patients (35% within group 18–57 y), while smaller proportions of older patients (28% within group >57 y) reported >3 conditions in comparison to younger patients (38% within group 18–57 y) (Table 2).

### CS-Base Items

The most frequently reported items that were problematic were “pain” (60%) and “fatigue” (58%). “Cognition” was the least reported, at 18% (SDC 3, Supplemental Digital Content 3, http://links.lww.com/MLR/C707). Compared with other conditions, patients with mental health problems reported that they experienced problems on almost all items. Items they reported most frequently were “mood” (72%), “anxiety” (85%), “self-esteem” (79%), and “fatigue” (77%). In contrast, diabetes patients reported problems on almost all items the least often compared with other conditions. The number of respondents reporting problems on the CS-Base items were consistent with the distribution of health conditions in our study. For example, “hearing” and “vision” were often reported among patients suffering from the condition of hearing or vision loss.

### Health-state Values

**FIGURE 2:**
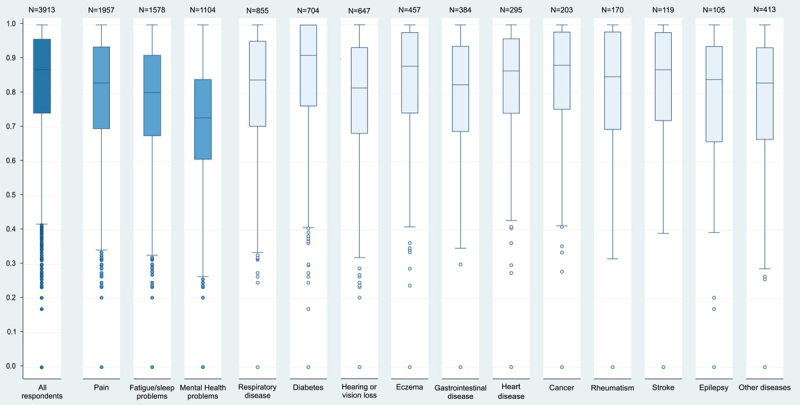
Boxplot of values for all respondents and each separate health condition (single health condition with or without comorbidity).

A total of 2436 different CS-Base health states were reported by patients in this study. The full health state (111111111111, value=1.0) was reported by 647 (17%) patients. The worst health state (444444444444, value=0.0) was reported by 2 patients. The mean value of the total sample was 0.83. The interquartile range of values for all respondents and for each separate condition were all between 0.60 and 1.0 (Fig. 2). Most of the respondents reported mild health states.

Males reported higher mean values (better health status) than females (*P*<0.001). Respondents in older age groups (>57 y) reported higher mean values than younger groups (*P*<0.001). Higher educated respondents also reported higher mean values than lower educated respondents (*P*<0.001, Table 1).

**FIGURE 3:**
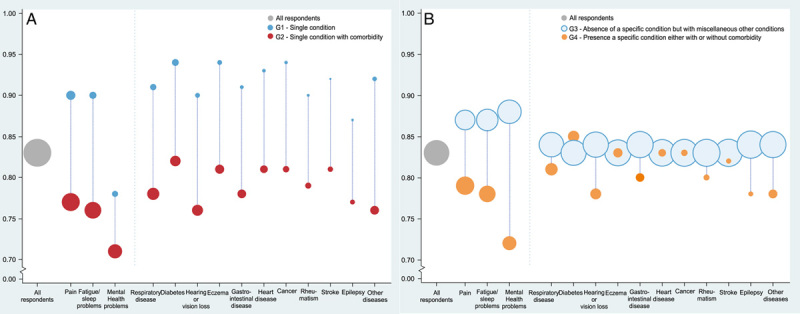
Distribution of mean Château Santé-Base values for each separate condition in 4 groups. A, G1—Single condition. G2—Single condition (as in G1) with comorbidity. B, G3—Absence of a specific condition (as in G1) but with miscellaneous other conditions. G4—Presence of a specific condition (as in G1) either with or without comorbidity. The size of the dots indicates the number of respondents of each separate condition. The 4 groups are different groups but not exclusive to each other, only G3 and G4 are mutually exclusive groups, G2 can be considered a subset of G4.

Among all 14 conditions, patients with diabetes and mental health problems reported the highest and lowest mean values (Figs. 2, 3: G1/G2/G4). Reversely, in group G3, patients who were absent from diabetes reported the lowest value, those absence of mental health problems reported the highest value.

Comorbidity was associated with reduced health status in our study. For all conditions, the mean values of group G1 were higher than G2 (Fig. 3), *P*<0.02 for all conditions (SDC 4, Supplemental Digital Content 4, http://links.lww.com/MLR/C708). The group G2 had lower mean values than G4, under the condition that both groups have a specific single condition.

The mean health-state values decreased when the number of conditions increased (*r*=−0.425, *P*<0.001), they were 0.90, 0.84, 0.79, 0.74, 0.71, and 0.67 for 1, 2, 3, 4, 5, and >5 conditions, respectively (Table 1). Comparing between combinations of multimorbidity (within top 5, N≥30), those included diabetes and mental health problems showed the highest and lowest mean values (Fig. 1). The second lowest mean values were observed in combinations of multimorbidity with coexisting fatigue/sleep problems and respiratory diseases.

## DISCUSSION

This study revealed that the CS-Base-reflected patients’ health conditions well, indicated by the fact that the number of observations of problems reported on the CS-Base items were consistent with the complaints that can be expected when looking at the distribution of health conditions reported. Such results would be expected from a measure that is fully patient-centered in its construction and development, and it is in line with the nature of patient-reported outcome measurement.

In accordance with other studies, we found that being male,^17,18^ and having higher education,^19,20^ were related to better health status. Generally, people’s health status deteriorates with aging, as studies have shown.^21,22^ In contrast, our study showed that values were higher with increasing age. One reason for this may be that the older patients in our study had less multimorbidity, which may indicate a better health condition than younger patients. Another reason may be related to older patients having a higher level of education compared with the younger patients in our study, as higher education has been known to be related to better health status.^23^ This is related to a limitation of our study. Our sample was representative regarding age and sex, but we did not deliberately seek national representativeness regarding education.

In this study, the worst health status was reported by patients with mental health problems, the best by patients with diabetes. Similar findings have been revealed by other studies. Regarding mental health problems, a German study investigated health status of general practice patients and found that depression had a stronger negative impact on health status than other diseases such as hypertension and diabetes.^24^ Another study evaluated the influence of emotional problems on health status and found that patients with emotional problems experienced reduced health status in all SF-36 domains.^25^ Regarding diabetes, a study in Iran showed that patients with diabetes reported the best health compared with those suffering from other chronic diseases including chronic renal disease, respiratory disease, hypertension, cancer, measured by the World Health Organization Quality-of-Life Scale (WHOQOL-BREF) questionnaire.^26^ A study measuring health status of diabetes patients in Portugal revealed the diabetes patients reporting quite positive health status.^27^

Corresponding with previous studies, our study also revealed that patients reported worse health status in the case of multimorbidity,^28,29^ or comorbidity.^30,31^ The impact of multimorbidity on health status varies depending on the different health conditions involved.^32^ According to our study, multimorbidity involving mental health problems revealed worse self-reported health status than multimorbidity involving other conditions. This is consistent with our finding that mental health problems as a separate health condition showed the worst self-reported health status. In addition, the coexistence of respiratory diseases and fatigue/sleep problems has been shown to be associated with worse self-reported health status, although to a lesser extent than multimorbidity involving mental health problems. The reason for this might be that patients with respiratory diseases combined with fatigue/sleep problems are at a severe stage of their disease. For example, compared with some mild respiratory diseases, chronic obstructive pulmonary disease (COPD) patients often suffer from severe symptoms such as shortness of breath, accompanied by fatigue.^33^ Their fatigue entails a daily lack of energy, lethargy, brain fog, weakness, heaviness, and tiredness, which cannot be relieved by sleep, and can substantially reduce patients’ health status.^34^ Our finding matches that of a previous study, in which researchers investigated the health status of >5000 patients with respiratory diseases involving asthma, allergic rhinitis, COPD, and rhinosinusitis. They found that a primary diagnosis of COPD having the worst impact on health status compared with other types of respiratory diseases.^35^

Our study was innovative in several aspects. The outcome measure used is preference-based, it assigns weights to health domains and measures the overall health status as a single metric value. This value allows for direct comparisons across different health conditions that may have impact on different health dimensions.^36^ Furthermore, this study places a strong emphasis on patient-centeredness. Not only is the content of the outcome measure fully based on patients’ selection, but also its values are generated based on patients’ response.

Regarding the trend toward patient involvement in health care, we see prospects of applying patient-centered, preference-based ePROMs to benefit various stakeholders including patients, clinicians, researchers, and policymakers. By using ePROMs, patients can be more empowered to monitor their own health status. Clinicians can track patients’ disease progression and tailor timely interventions. Shared decision-making for treatment involving both clinicians and patients can also be achieved. Researchers can conduct more efficient patient-centered studies regarding the health status of populations. Policymakers ultimately can get access to more sources and robust evidence to make policies for improving quality of health care.

## Supplementary Material

SUPPLEMENTARY MATERIAL
