# Supplementary material for: Health Status of US Patients With One or More Health Conditions: Using a Novel Electronic Patient-reported Outcome Measure Producing Single Metric Measures
Source: Med Care. 2023 Sep 13;61(11):765–71. doi: 10.1097/MLR.0000000000001919 (PMC10563950; doi:10.1097/MLR.0000000000001919)

**Figure A1.1**

Screenshot of CS-Base as depicted in the HealthSnApp (an application for mobile phones): 12 items, each with 4 levels


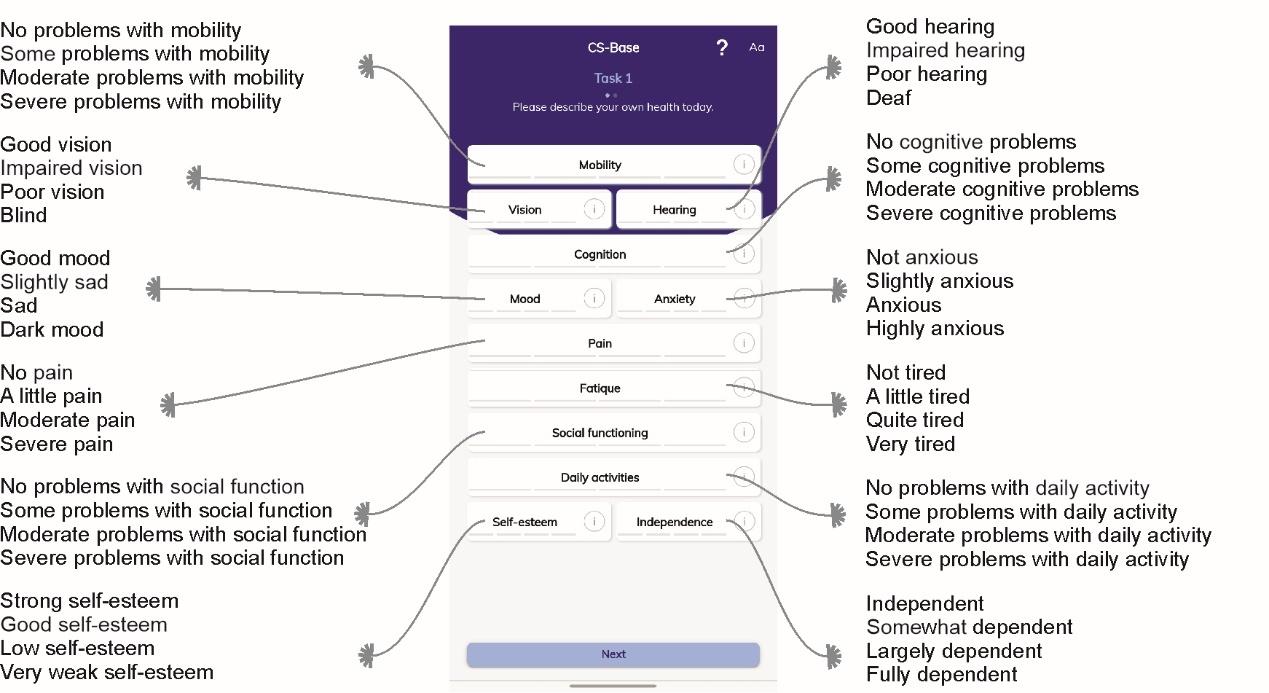


**Figure A1.2**

Screenshots of the CS-Base from the HealthSnApp depicting Task 1.

In this descriptive task, all health items were listed in interactive (rotating) boxes presented on a single screen. When a patient selected the interactive box for a specific item, the box displayed response options. For example, when a patient selected the “Fatigue” box, the display shifted to offer the following response options: “Not tired,” “A little tired,” “Quite tired,” and “Very tired.”


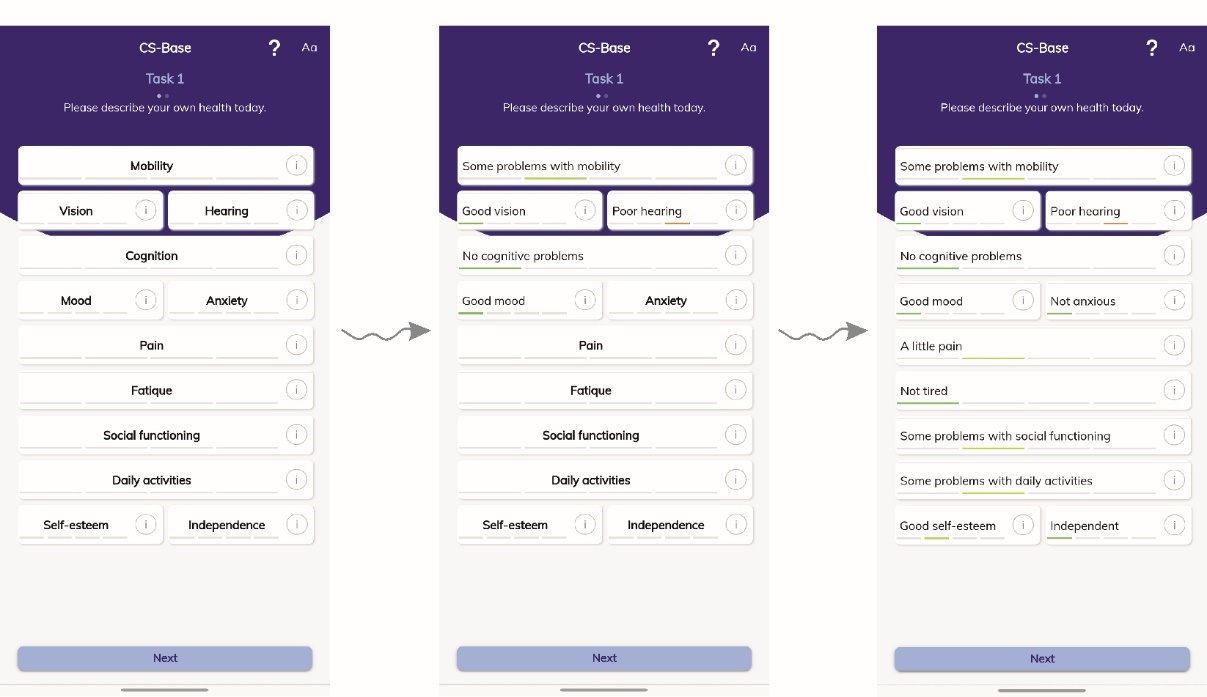


**Figure A1.3**

Screenshots from the HealthSnApp depicting the use of the Drop-Down (DD) method.

Patients first assessed and described their current health status under Task 1. They were subsequently directed to Task 2 (DD method). In Task 2, their health states, assessed in Task 1, were presented (Figure A1.3-**A**). They then made multiple selections (1–5 times) of items that disturbed them most and dropped down to one level lower by swiping it. For example, the patient dropped down to one level lower for the “hearing” item for the first of the selections (Figure A1.3-B).


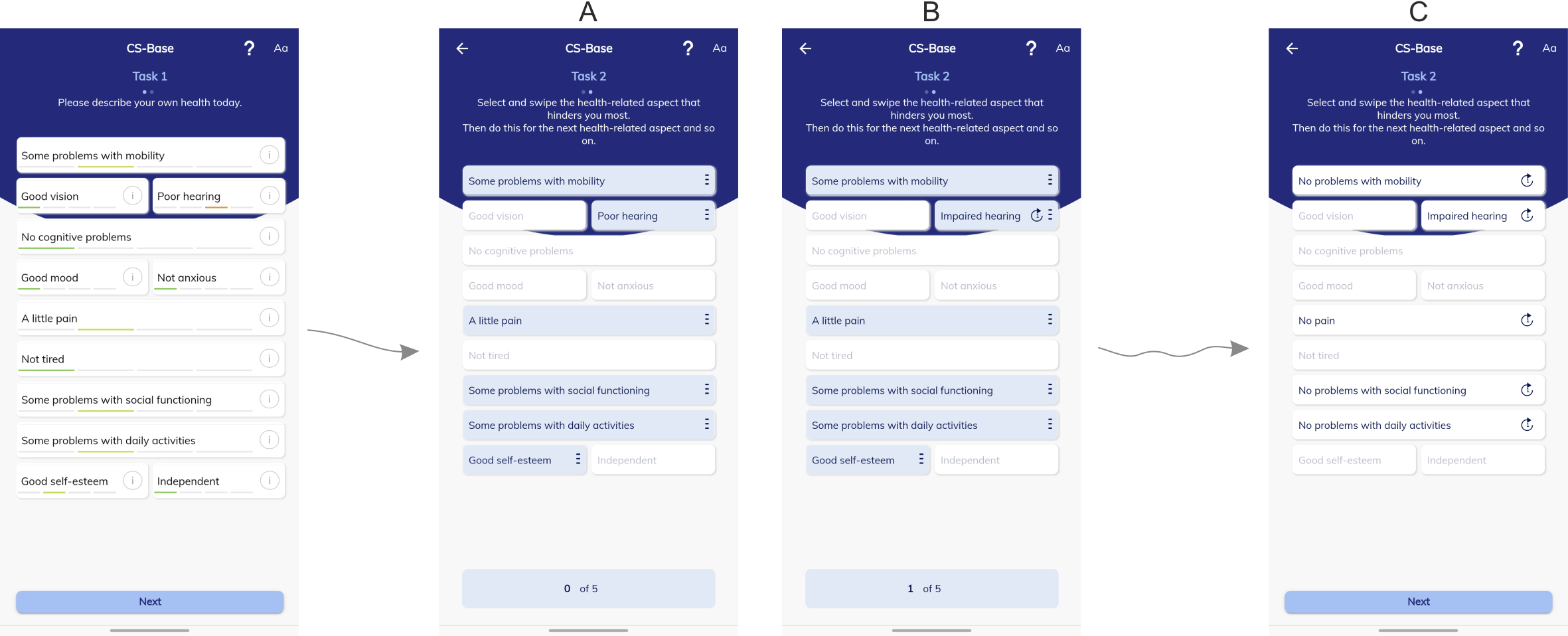

Supplement: SUPPLEMENTARY MATERIAL [file mlr-61-765-s001.docx]
