# Supplementary material for: Health Status of US Patients With One or More Health Conditions: Using a Novel Electronic Patient-reported Outcome Measure Producing Single Metric Measures
Source: Med Care. 2023 Sep 13;61(11):765–71. doi: 10.1097/MLR.0000000000001919 (PMC10563950; doi:10.1097/MLR.0000000000001919)
Supplement: SUPPLEMENTARY MATERIAL [file mlr-61-765-s002.docx]

Computing of CS-Base health-state value

Raw values for health states were first computed in STATA, based on coefficients (weights) of each level of all the CS-Base items (Table A1), using the formula:

Raw Values=

Coef_A12*iA12 + Coef_A13*iA13 + Coef_A14*iA14 +

Coef_A22*iA22 + Coef_A23*iA23 + Coef_A24*iA24 +

Coef_A32*iA32 + Coef_A33*iA33 + Coef_A24*iA34 +

Coef_A42*iA42 + Coef_A43*iA43 + Coef_A24*iA44 +

Coef_A52*iA52 + Coef_A53*iA53 + Coef_A24*iA54 +

Coef_A62*iA62 + Coef_A63*iA63 + Coef_A24*iA64 +

Coef_A72*iA72 + Coef_A73*iA73 + Coef_A24*iA74 +

Coef_A82*iA82 + Coef_A83*iA83 + Coef_A24*iA84 +

Coef_A92*iA92 + Coef_A93*iA93 + Coef_A24*iA94 +

Coef_A102*iA102 + Coef_A103*iA103 + Coef_A24*iA104 +

Coef_A112*iA112 + Coef_A113*iA113 + Coef_A24*iA114 +

Coef_A122*iA122 + Coef_A123*iA123 + Coef_A24*iA124

- Coef_A12=coefficient of Level 2 of the first item, and so on for other levels of each item
- iA12=dummy of Level 2 of the first item, and so on for other levels of each item

Raw values were rescaled to 0.0 to 1.0 using the formula:

zi=(xi–min(x))/(max(x)–min(x))

- zi indicated the i^th^ rescaled value
- xi indicated the i^th^ raw value,
- min(x) indicates the minimum raw value (=−158.76 in this study, worst health state 444444444444)
- max(x) indicates the maximum raw value (=0.0 in this study, full health state 111111111111)

Table A1 Coefficients of each level of all the CS-Base items

| **CS-Base Items levels** | **Coefficient** |
| --- | --- |
| Mobility (2) | ˗3.22 |
| Mobility (3) | ˗8.95 |
| Mobility (4) | ˗15.40 |
|  |  |
| Pain (2) | ˗3.23 |
| Pain (3) | ˗7.54 |
| Pain (4) | ˗13.14 |
|  |  |
| Anxiety (2) | ˗3.13 |
| Anxiety (3) | ˗7.44 |
| Anxiety (4) | ˗12.94 |
|  |  |
| Daily activity (2) | ˗3.46 |
| Daily activity (3) | ˗7.65 |
| Daily activity (4) | ˗11.72 |
|  |  |
| Cognition (2) | ˗3.28 |
| Cognition (3) | ˗8.19 |
| Cognition (4) | ˗12.87 |
|  |  |
|  |  |
| Mood (2) | ˗3.30 |
| Mood (3) | ˗7.89 |
| Mood (4) | ˗13.19 |
|  |  |
| Fatigue (2) | ˗3.40 |
| Fatigue (3) | ˗7.65 |
| Fatigue (4) | ˗12.55 |
|  |  |
| Vision (2) | ˗3.25 |
| Vision (3) | ˗8.24 |
| Vision (4) | ˗14.55 |
|  |  |
| Hearing (2) | -3.45 |
| Hearing (3) | ˗8.66 |
| Hearing (4) | ˗14.76 |
|  |  |
| Social function (2) | ˗3.44 |
| Social function (3) | ˗7.56 |
| Social function (4) | -12.71 |
|  |  |
| Self-esteem (2) | ˗3.81 |
| Self-esteem (3) | ˗7.54 |
| Self-esteem (4) | ˗12.45 |
|  |  |
| Independence (2) | -3.83 |
| Independence (3) | ˗8.15 |
| Independence (4) | -12.50 |
