# Supplementary material for: Health Status of US Patients With One or More Health Conditions: Using a Novel Electronic Patient-reported Outcome Measure Producing Single Metric Measures
Source: Med Care. 2023 Sep 13;61(11):765–71. doi: 10.1097/MLR.0000000000001919 (PMC10563950; doi:10.1097/MLR.0000000000001919)
Supplement: SUPPLEMENTARY MATERIAL [file mlr-61-765-s003.docx]

**Table A2**

The number of respondents reported on each health condition and each level of all the CS-Base items

| **CS-Base items and levels N (%)** | **Health conditions N (%)** | | | | | | | | | | | | | | |
| --- | --- | --- | --- | --- | --- | --- | --- | --- | --- | --- | --- | --- | --- | --- | --- |
|  | All respondents | Pain | Fatigue/ sleep problems | Mental health problems | Respiratory diseases | Diabetes | Hearing or vision loss | Eczema | Gastrointestinal disease | Heart disease | Cancer | Rheumatism | Stroke | Epilepsy | Other diseases |
|  | 3913 (100) | 1957 (50) | 1578 (40) | 1104 (18) | 855 (22) | 704 (18) | 647 (17) | 457 (12) | 384 (10) | 295 (8) | 203 (5) | 170 (4) | 119 (3) | 105 (3) | 413 (11) |
| Mobility |  |  |  |  |  |  |  |  |  |  |  |  |  |  |  |
| No problems | 2807 (72) | 1207 (62) | 1048 (66) | 700 (63) | 541 (63) | 477 (68) | 401 (62) | 344 (75) | 231 (60) | 166 (56) | 124 (61) | 97 (57) | 63 (53) | 69 (66) | 244 (59) |
| Some problems | 807 (21) | 524 (27) | 363 (23) | 273 (25) | 212 (25) | 154 (22) | 166 (27) | 81 (18) | 99 (26) | 90 (31) | 56 (28) | 47 (28) | 37 (31) | 24 (23) | 106 (26) |
| Moderate problems | 212 (5) | 162 (8) | 112 (7) | 93 (8) | 73 (9) | 56 (8) | 62 (10) | 24 (5) | 35 (9) | 26 (9) | 15 (7) | 15 (9) | 12 (10) | 10 (10) | 39 (9) |
| Severe problems | 87 (2) | 64 (3) | 55 (3) | 38 (3) | 29 (3) | 17 (2) | 18 (3) | 8 (2) | 19 (5) | 13 (4) | 8 (4) | 11 (6) | 7 (6) | 2 (2) | 24 (6) |
| Vision |  |  |  |  |  |  |  |  |  |  |  |  |  |  |  |
| Good | 3041 (78) | 1491 (76) | 1178 (75) | 820 (74) | 642 (75) | 540 (77) | 252 (39) | 381 (83) | 291 (76) | 204 (69) | 153 (75) | 128 (75) | 89 (75) | 80 (76) | 302 (73) |
| Impaired | 725 (19) | 382 (20) | 330 (21) | 225 (20) | 175 (20) | 128 (18) | 306 (47) | 62 (14) | 76 (20) | 75 (25) | 44 (22) | 33 (19) | 26 (22) | 19 (18) | 92 (22) |
| Poor | 127 (3) | 75 (4) | 64 (4) | 48 (4) | 32 (4) | 28 (4) | 77 (12) | 12 (3) | 14 (4) | 12 (4) | 4 (2) | 7 (4) | 2 (2) | 3 (3) | 16 (4) |
| Blind | 20 (1) | 9 (0) | 6 (0) | 11 (1) | 6 (1) | 8 (1) | 12 (2) | 2 (0) | 3 (1) | 4 (1) | 2 (1) | 2 (1) | 2 (2) | 3 (3) | 3 (1) |
| Hearing |  |  |  |  |  |  |  |  |  |  |  |  |  |  |  |
| Good | 2191 (56) | 1023 (52) | 774 (49) | 457 (41) | 438 (51) | 443 (63) | 211 (33) | 270 (59) | 185 (48) | 170 (58) | 113 (56) | 96 (56) | 68 (57) | 49 (47) | 181 (44) |
| Impaired | 1403 (36) | 729 (37) | 630 (40) | 497 (45) | 337 (40) | 218 (31) | 330 (51) | 144 (32) | 166 (43) | 105 (36) | 75 (37) | 58 (34) | 43 (36) | 41 (39) | 191 (46) |
| Poor | 311 (8) | 198 (10) | 170 (11) | 145 (13) | 77 (9) | 41 (6) | 101 (16) | 40 (9) | 30 (8) | 18 (6) | 13 (6) | 14 (8) | 7 (6) | 14 (13) | 40 (10) |
| Deaf | 8 (0) | 7 (0) | 4 (0) | 5 (0) | 3 (0) | 2 (0) | 5 (1) | 3 (1) | 3 (1) | 2 (1) | 2 (1) | 2 (1) | 1 (1) | 1 (1) | 1 (0) |
| Cognition |  |  |  |  |  |  |  |  |  |  |  |  |  |  |  |
| No problems | 3203 (82) | 1519 (78) | 1190 (75) | 748 (69) | 656 (77) | 567 (81) | 475 (73) | 383 (84) | 292 (76) | 224 (76) | 161 (79) | 130 (76) | 83 (70) | 67 (64) | 307 (74) |
| Some problems | 581 (15) | 358 (18) | 321 (20) | 275 (25) | 156 (18) | 109 (15) | 135 (21) | 56 (12) | 68 (18) | 57 (19) | 34 (17) | 29 (17) | 29 (24) | 33 (31) | 83 (20) |
| Moderate problems | 101 (3) | 60 (3) | 54 (3) | 61 (6) | 32 (4) | 19 (3) | 27 (4) | 13 (3) | 16 (4) | 11 (4) | 5 (2) | 8 (5) | 4 (3) | 3 (3) | 18 (4) |
| Severe problems | 28 (1) | 20 (1) | 13 (1) | 20 (2) | 11 (1) | 9 (1) | 10 (2) | 5 (1) | 8 (2) | 3 (1) | 3 (1) | 3 (2) | 3 (3) | 2 (2) | 5 (1) |
| Mood |  |  |  |  |  |  |  |  |  |  |  |  |  |  |  |
| Good | 2274 (58) | 1040 (53) | 701 (44) | 300 (28) | 468 (55) | 467 (66) | 348 (54) | 267 (58) | 201 (52) | 192 (65) | 124 (61) | 99 (58) | 74 (62) | 50 (48) | 210 (51) |
| Slightly sad | 1004 (26) | 531 (27) | 490 (31) | 388 (35) | 235 (28) | 148 (21) | 173 (27) | 118 (26) | 112(29) | 67 (23) | 52 (26) | 43 (25) | 31 (26) | 29 (28) | 121 (29) |
| Sad | 440 (11) | 261 (13) | 260 (16) | 277 (25) | 98 (11) | 63 (9) | 82 (13) | 39 (9) | 50 (13) | 20 (7) | 17 (8) | 18 (11) | 7 (6) | 15 (14) | 58 (14) |
| Dark | 195 (5) | 125 (6) | 127 (8) | 139 (13) | 54 (6) | 26 (4) | 44 (7) | 33 (7) | 21 (5) | 16 (5) | 10 (5) | 10 (6) | 7 (6) | 11 (10) | 24 (6) |
| Anxiety |  |  |  |  |  |  |  |  |  |  |  |  |  |  |  |
| Not anxious | 1813 (46) | 794 (41) | 496 (31) | 160 (15) | 356 (42) | 410 (58) | 269 (42) | 206 (45) | 157 (41) | 148 (50) | 109 (54) | 84 (49) | 63 (53) | 36 (34) | 178 (43) |
| Slightly anxious | 1117 (29) | 595 (30) | 512 (32) | 336 (30) | 256 (30) | 165 (23) | 199 (31) | 116 (25) | 122 (32) | 84 (28) | 53 (26) | 43 (25) | 30 (25) | 33 (31) | 113 (27) |
| Anxious | 637 (16) | 375 (19) | 359 (23) | 351 (32) | 138 (16) | 86 (12) | 105 (16) | 88 (19) | 65 (17) | 41 (14) | 24 (12) | 29 (17) | 19 (16) | 19 (18) | 72 (17) |
| Highly anxious | 346 (9) | 193 (10) | 211 (13) | 257 (23) | 105 (12) | 43 (4) | 74 (11) | 47 (10) | 40 (10) | 22 (7) | 17 (8) | 14 (8) | 7 (6) | 17 (16) | 50 (12) |
| Pain |  |  |  |  |  |  |  |  |  |  |  |  |  |  |  |
| No | 1561 (40) | 435 (22) | 500 (32) | 341 (31) | 317 (37) | 321 (46) | 195 (30) | 211 (46) | 112 (29) | 108 (37) | 75 (37) | 59 (35) | 46 (39) | 42 (40) | 111 (27) |
| A little | 1292 (33) | 689 (35) | 542 (34) | 342 (31) | 240 (28) | 191 (27) | 230 (36) | 139 (30) | 120 (31) | 85 (29) | 66 (33) | 38 (22) | 30 (25) | 23 (22) | 138 (33) |
| Moderate | 791 (20) | 603 (31) | 385 (24) | 295 (27) | 209 (25) | 131 (19) | 154 (24) | 83 (18) | 106 (28) | 78 (26) | 41 (20) | 49 (29) | 36 (30) | 28 (27) | 111 (27) |
| Severe | 269 (7) | 230 (12) | 151 (10) | 126 (11) | 89 (10) | 61 (9) | 68 (11) | 24 (5) | 46 (12) | 24 (8) | 21 (10) | 24 (14) | 7 (6) | 12 (11) | 53 (13) |
| Fatigue |  |  |  |  |  |  |  |  |  |  |  |  |  |  |  |
| Not tired | 1628 (42) | 646 (33) | 362 (23) | 251 (23) | 326 (38) | 342 (49) | 212 (33) | 201 (44) | 123 (32) | 125 (42) | 86 (42) | 71 (42) | 58 (49) | 37 (35) | 129 (31) |
| A little tired | 1245 (32) | 658 (34) | 543 (34) | 347 (31) | 242 (28) | 217 (31) | 224 (35) | 132 (29) | 138 (36) | 98 (33) | 67 (33) | 46 (27) | 33 (28) | 32 (30) | 133 (32) |
| Quite tired | 780 (20) | 486 (25) | 482 (31) | 345 (31) | 208 (24) | 111 (16) | 148 (23) | 86 (19) | 83 (22) | 50 (17) | 36 (18) | 34 (20) | 20 (17) | 25 (24) | 99 (24) |
| Very tired | 260 (7) | 167 (9) | 190 (12) | 161 (15) | 79 (9) | 34 (5) | 63 (10) | 38 (8) | 40 (10) | 22 (7) | 14 (7) | 19 (11) | 8 (7) | 11 (10) | 52 (13) |
| Social function |  |  |  |  |  |  |  |  |  |  |  |  |  |  |  |
| No problems | 2472 (63) | 1138 (58) | 813 (52) | 362 (33) | 509 (60) | 493 (70) | 368 (57) | 281 (61) | 226 (59) | 184 (62) | 132 (65) | 98 (58) | 68 (57) | 53 (50) | 236 (57) |
| Some problems | 903 (23) | 491 (25) | 454 (29) | 385 (35) | 204 (24) | 144 (20) | 164 (25) | 112 (25) | 95 (25) | 72 (24) | 48 (24) | 45 (26) | 31 (26) | 26 (25) | 107 (26) |
| Moderate problems | 357 (9) | 223 (11) | 194 (12) | 221 (20) | 95 (11) | 42 (6) | 76 (12) | 42 (9) | 42 (11) | 21 (7) | 11 (5) | 23 (14) | 17 (14) | 15 (14) | 38 (9) |
| Severe problems | 181 (5) | 105 (5) | 117 (7) | 136 (12) | 47 (6) | 25 (4) | 39 (6) | 22 (5) | 21 (5) | 18 (6) | 12 (6) | 4 (2) | 3 (3) | 11 (10) | 32 (8) |
| Daily activity |  |  |  |  |  |  |  |  |  |  |  |  |  |  |  |
| No problems | 2449 (63) | 1051 (54) | 825 (52) | 456 (41) | 471 (55) | 455 (65) | 356 (55) | 296 (65) | 197 (51) | 165 (56) | 116 (57) | 82 (48) | 63 (53) | 59 (56) | 205 (50) |
| Some problems | 1045 (27) | 611 (31) | 523 (33) | 408 (37) | 259 (30) | 164 (23) | 193 (30) | 112 (25) | 115 (30) | 88 (30) | 63 (31) | 53 (31) | 29 (24) | 31 (30) | 137 (33) |
| Moderate problems | 326 (8) | 230 (12) | 181 (11) | 180 (16) | 99 (12) | 67 (10) | 73 (11) | 36 (8) | 59 (15) | 32 (11) | 16 (8) | 27 (16) | 23 (19) | 9 (9) | 53 (13) |
| Severe problems | 93 (2) | 65 (3) | 49 (3) | 60 (5) | 26 (3) | 18 (3) | 25 (4) | 13 (3) | 13 (3) | 10 (3) | 8 (4) | 8 (5) | 4 (3) | 6 (6) | 18 (4) |
| Self-esteem |  |  |  |  |  |  |  |  |  |  |  |  |  |  |  |
| Strong | 1930 (49) | 867 (44) | 554 (35) | 233 (21) | 391 (46) | 423 (60) | 276 (43) | 228 (50) | 171 (45) | 164 (56) | 119 (59) | 87 (51) | 71 (60) | 52 (50) | 163 (39) |
| Good | 835 (21) | 410 (21) | 355 (23) | 217 (20) | 180 (21) | 126 (18) | 149 (23) | 88 (19) | 72 (19) | 59 (20) | 42 (21) | 35 (21) | 15 (13) | 15 (14) | 113 (27) |
| Low | 833 (21) | 498 (25) | 468 (30) | 433 (39) | 215 (25) | 114 (16) | 161 (25) | 102 (22) | 101 (26) | 53 (18) | 26 (13) | 34 (20) | 24 (20) | 26 (25) | 91 (22) |
| Very weak | 315 (8) | 182 (9) | 201 (13) | 221 (20) | 69 (8) | 41 (6) | 61 (9) | 39 (9) | 40 (10) | 19 (6) | 16 (8) | 14 (8) | 9 (8) | 12 (11) | 46 (11) |
| Independence |  |  |  |  |  |  |  |  |  |  |  |  |  |  |  |
| Independent | 2915 (75) | 1389 (71) | 1052 (67) | 604 (55) | 608 (71) | 559 (79) | 447 (69) | 330 (72) | 279 (73) | 219 (74) | 154 (76) | 123 (72) | 84 (71) | 71 (68) | 278 (67) |
| Somewhat dependent | 733 (19) | 420 (21) | 382 (24) | 334 (30) | 187 (22) | 107 (15) | 145 (22) | 86 (19) | 76 (20) | 57 (19) | 37 (18) | 32 (19) | 22 (18) | 23 (22) | 103 (25) |
| Largely dependence | 206 (5) | 116 (6) | 108 (7) | 128 (12) | 44 (5) | 27 (4) | 42 (7) | 28 (6) | 22 (6) | 15 (5) | 8 (4) | 10 (6) | 9 (8) | 9 (9) | 24 (6) |
| Fully dependent | 59 (2) | 32 (2) | 36 (2) | 38 (3) | 16 (2) | 11 (2) | 13 (2) | 13 (3) | 7 (2) | 4 (1) | 4 (2) | 5 (3) | 4 (3) | 2 (2) | 8 (2) |
