# Supplementary material for: Health Status of US Patients With One or More Health Conditions: Using a Novel Electronic Patient-reported Outcome Measure Producing Single Metric Measures
Source: Med Care. 2023 Sep 13;61(11):765–71. doi: 10.1097/MLR.0000000000001919 (PMC10563950; doi:10.1097/MLR.0000000000001919)
Supplement: SUPPLEMENTARY MATERIAL [file mlr-61-765-s004.docx]

**Table A3**

Mean CS-Base values for four groups of various conditions

| **Health conditions** | **G1-Single condition**  **(without comorbidities)** | | **G2-Single condition with comorbidities** | | **G3-Absence of a condition (without this condition but with miscellaneous other conditions)** | | **G4-Presence of a condition**  **with or without comorbidities** | | **P value (G1/G2)** |
| --- | --- | --- | --- | --- | --- | --- | --- | --- | --- |
|  | **N** | **Mean value (SD)** | **N** | **Mean value (SD)** | **N** | **Mean value (SD)** | **N** | **Mean value (SD)** |  |
| Pain | 369 | 0.90 (0.11) | 1588 | 0.77 (0.17) | 1956 | 0.87 (0.13) | 1957 | 0.79 (0.16) | <0.001 |
| Fatigue/sleep problems | 209 | 0.90 (0.11) | 1369 | 0.76 (0.16) | 2335 | 0.87 (0.14) | 1578 | 0.78 (0.16) | <0.001 |
| Mental health problems | 145 | 0.78 (0.14) | 959 | 0.71 (0.17) | 2809 | 0.88 (0.12) | 1104 | 0.72 (0.17) | <0.001 |
| Respiratory diseases | 159 | 0.91 (0.10) | 696 | 0.78 (0.17) | 3058 | 0.84 (0.15) | 855 | 0.81 (0.17) | <0.001 |
| Diabetes | 195 | 0.94 (0.10) | 509 | 0.82 (0.17) | 3209 | 0.83 (0.15) | 704 | 0.85 (0.17) | <0.001 |
| Hearing or vision loss | 85 | 0.90 (0.10) | 562 | 0.76 (0.18) | 3266 | 0.84 (0.15) | 647 | 0.78 (0.18) | <0.001 |
| Eczema | 86 | 0.94 (0.07) | 371 | 0.81 (0.18) | 3456 | 0.83 (0.15) | 457 | 0.83 (0.17) | <0.001 |
| Gastrointestinal diseases | 47 | 0.91 (0.11) | 337 | 0.78 (0.18) | 3529 | 0.84 (0.15) | 384 | 0.80 (0.17) | <0.001 |
| Heart disease | 40 | 0.93 (0.10) | 255 | 0.81 (0.17) | 3618 | 0.83 (0.15) | 295 | 0.83 (0.17) | <0.001 |
| Cancer | 35 | 0.94 (0.08) | 168 | 0.81 (0.19) | 3710 | 0.83 (0.15) | 203 | 0.83 (0.18) | <0.001 |
| Rheumatism | 21 | 0.90 (0.18) | 149 | 0.79 (0.20) | 3743 | 0.83 (0.15) | 170 | 0.80 (0.20) | 0.0164 |
| Stroke | 8 | 0.92 (0.07) | 111 | 0.81 (0.19) | 3794 | 0.83 (0.15) | 119 | 0.82 (0.18) | 0.005 |
| Epilepsy | 16 | 0.87 (0.12) | 89 | 0.77 (0.21) | 3808 | 0.84 (0.15) | 105 | 0.78 (0.20) | 0.010 |
| Other diseases | 73 | 0.92 (0.09) | 340 | 0.76 (0.18) | 3500 | 0.84 (0.15) | 413 | 0.78 (0.18) | <0.001 |

Explanation of groups using the example of Pain, G1 = patients with the single condition of pain, G2 = patients have pain and one or more other conditions, G3 = patients do not have the condition of pain but have miscellaneous other conditions, G4 = patients have pain either with or without miscellaneous other conditions.

The four groups are different groups but not exclusive to each other. Only the G3 and G4 are mutually exclusive groups. G2 can be considered a subset of G4.
